# Exploratory Metabolomic Profiling of Plasma and Cerebrospinal Fluid in a Pilot Study of Children with Acute Lymphoblastic Leukemia

**DOI:** 10.3390/cells15141255

**Published:** 2026-07-12

**Authors:** Andrzej Wasilewski, Hanna Czapor-Irzabek, Milena Ściskalska, Adam El Idrissi, Fatima Chegdani, Agnieszka Matera-Witkiewicz, Tomasz Zatoński, Katarzyna Połtyn-Zaradna, Tomasz Brutkowski, Aleksandra Klimczak, Bernarda Kazanowska, Agata Serrafi

**Affiliations:** 1 Student Scientific Association of Medical Chemistry and Immunochemistry, Wroclaw Medical University, ul. M. Skłodowskiej-Curie 48/50, 59-369 Wroclaw, Poland; 2 Laboratory of Elemental Analysis and Structural Research, Faculty of Pharmacy, Wroclaw Medical University, 50-556 Wroclaw, Poland; hanna.czapor-irzabek@umw.edu.pl; 3 Department of Pharmaceutical Biochemistry, Faculty of Pharmacy, Wroclaw Medical University, Borowska 211A, 50-556 Wroclaw, Poland; milena.sciskalska@umw.edu.pl; 4 Department of Animal Science, Food and Nutrition (DiANA), Università Cattolica del Sacro Cuore, Via Emilia Parmense, 84, 29122 Piacenza, PC, Italy; adam.elidrissi@unicatt.it; 5 Laboratory of Integrative Biology, Department of Biology, Faculty of Sciences Aïn Chock, Hassan II University of Casablanca, Route El Jadida, BP 5366 Maarif, Casablanca 20100, Morocco; fchegdani@yahoo.fr; 6 Screening of Biological Activity Assays and Collection of Biological Material Laboratory, Wroclaw Medical University Biobank, Faculty of Pharmacy, Wroclaw Medical University, 50-556 Wroclaw, Poland; agnieszka.matera-witkiewicz@umw.edu.pl; 7 Department and Clinic of Otolaryngology, Head and Neck Surgery, Wroclaw Medical University, Borowska 213, 50-556 Wroclaw, Poland; tomasz.zatonski@umw.edu.pl; 8 Department of Social Medicine, Wroclaw Medical University, 50-345 Wroclaw, Poland; katarzyna.poltyn-zarada@umw.edu.pl; 9 Department of Bone Marrow Transplantation, Pediatric Oncology and Hematology, Wroclaw Medical University, ul. Borowska 213, 50-556 Wroclaw, Poland; tomasz.brutkowski@umw.edu.pl (T.B.); bernarda.kazanowska@umw.edu.pl (B.K.); 10 Laboratory of Biology of Stem and Neoplastic Cells, Hirszfeld Institute of Immunology and Experimental Therapy, Polish Academy of Sciences, R. Weigla 12, 53-114 Wroclaw, Poland; aleksandra.klimczak@hirszfeld.pl; 11 Division of Medical Biochemistry, Department of Biochemistry and Immunochemistry, Faculty of Medicine, Wroclaw Medical University, ul. Chałubińskiego 10, 50-369 Wroclaw, Poland

**Keywords:** acute lymphoblastic leukemia, metabolic profiling, pediatric oncology, mass spectrometry, metabolomics

## Abstract

**Highlights:**

**What are the main findings?**

- Treatment-naive children with pre-B ALL appear to show a distinct plasma metabolic signature, suggesting early disruptions in purine, lipid, and amino acid pathways.
- Altered levels of purine catabolites (such as hypoxanthine and xanthine) in the systemic circulation seem to be reflected within the cerebrospinal fluid (CSF) microenvironment.

**What are the implications of the main findings?**

- The observed correlation between plasma and CSF suggests that non-invasive blood metabolomics may have the potential to indirectly monitor central nervous system involvement. However, this preliminary observation warrants further clinical validation.
- This exploratory baseline profile may serve as a proof-of-concept to guide future large-scale biomarker discovery and personalized therapy development in pediatric oncology.

**Abstract:**

Acute lymphoblastic leukemia (ALL) is the most common pediatric malignancy and is associated with profound metabolic reprogramming. This exploratory study aimed to characterize the metabolomic profiles of plasma and cerebrospinal fluid (CSF) in children with newly diagnosed pre-B-cell acute lymphoblastic leukemia (pre-B ALL) prior to therapy. Metabolomic analyses were performed using mass spectrometry-based platforms combined with multivariate statistical approaches (PCA, OPLS-DA, SVM-RFE, EBAM). In plasma, we identified 41 significantly altered metabolites (FDR < 0.011), revealing a distinct signature that differentiated pre-B ALL patients from healthy controls. Specifically, patients exhibited elevated levels of hypoxanthine, xanthine, and phosphatidylcholine derivatives, alongside reduced concentrations of *L*-cysteine and prasterone sulfate, indicating systemic dysregulation of purine, lipid, and amino acid metabolism. In CSF, we observed a distinct metabolic profile characterized by coordinated disturbances in purine degradation, phospholipid metabolism, and sphingolipid pathways. Notably, correlation analysis between the two matrices suggested that systemic metabolic shifts, particularly in purine metabolism (e.g., hypoxanthine and xanthine levels), are mirrored within the central nervous system microenvironment. These findings indicate that children with pre-B ALL exhibit specific metabolic alterations in both compartments before treatment. This work serves as a proof-of-concept for applying metabolomics in pediatric oncology, highlighting the necessity for further validation in larger, prospective cohorts to assess the clinical utility of these profiles.

## 1. Introduction

Acute lymphoblastic leukemia (ALL) and acute myeloid leukemia (AML) account for approximately 80% and 15–20% of childhood leukemias, respectively, making them the most prevalent pediatric cancers [1]. Although contemporary treatment protocols allow for cure rates exceeding 85% for ALL, significant challenges persist, including treatment resistance, relapse, and long-term therapy-related complications, such as neurodevelopmental and metabolic disorders [2,3]. Understanding the molecular mechanisms underlying leukemia pathogenesis and treatment response is essential for improving prognosis and minimizing therapeutic toxicity.

In recent years, metabolomics defined as the comprehensive analysis of small-molecule metabolites in biological systems has become a key tool in oncological research, complementing genomics and proteomics data [4]. As the downstream products of cellular processes, metabolites provide a direct reflection of the cellular phenotype and a functional record of the disease state or response to therapeutic interventions. In the context of pediatric leukemias, metabolic profiling enables the identification of diagnostic biomarkers, monitoring of treatment response, and assessment of therapy-related metabolic complications [5,6]. Due to their high sensitivity and specificity, mass spectrometry (MS)-based techniques allow for the detailed analysis of complex biological matrices, such as plasma or bone marrow using minimal sample volumes, which is particularly critical in pediatric studies [7,8].

Acute lymphoblastic leukemia (ALL) is a hematological malignancy characterized by the abnormal proliferation of lymphoid progenitor cells, primarily in the bone marrow, with possible involvement of extramedullary sites such as the central nervous system (CNS) [9]. Despite major therapeutic advances, ALL remains biologically and clinically complex, supporting the need for complementary approaches, including integrative approaches, or different timepoints studies or possibly a multi-omic integration from another perspective [10,11]. Previous metabolomic studies have reported alterations in glycerophospholipid and fatty acid metabolism in ALL serum, suggesting that lipid dysregulation may reflect increased membrane turnover and cellular proliferation in leukemic cells [12]. This has been verified by different studies which have consistently reported metabolic dysregulation [13]. In addition to its association with increased membrane turnover, dysregulation of glycerophospholipid metabolism may reflect alterations in membrane-associated signaling processes in leukemic cells [14]. Glycerophospholipids constitute structural components of the plasma membrane, while phosphatidylinositol-derived intermediates, including PIP2 and PIP3, function as important mediators of B-cell activation signaling [15]. In lymphoid malignancies, disruption of this lipid environment may facilitate sustained B-cell receptor-related signaling and activation of downstream PI3K/AKT pathways, which are often activated by genomic mutations [15,16]. Accordingly, the enrichment of glycerophospholipid metabolism observed in our analysis may indicate possible membrane remodeling or altered activity of oncogenic metabolic signals in sick patients.

Despite the growing number of studies, data concerning specific metabolic signatures in children with leukemia, particularly those involving simultaneous analysis of plasma immediately at the time of diagnosis, remain limited. The identification of early biochemical markers may, in the future, significantly support risk stratification and enable the personalization of oncological therapy.

The primary objective of this study was to conduct a comprehensive analysis of the metabolic profile of plasma in children with newly diagnosed pre-B ALL. To eliminate the impact of pharmacotherapy on patient metabolism, biological samples were collected during standard diagnostic procedures, prior to the initiation of anticancer treatment. These metabolic profiles were compared with those of a demographically matched (by age and sex) control group of healthy children, allowing for the precise identification of biochemical pathways disrupted during the neoplastic process.

## 2. Materials and Methods

### 2.1. Study Design

The study cohort consisted of 19 pediatric patients (8 females, 11 males; median age 4.75 years; range: 2–14 years) with a new diagnosis of pre-B acute lymphoblastic leukemia (ALL). From these patients, both peripheral blood (plasma) and cerebrospinal fluid (CSF) samples were collected during standard diagnostic procedures prior to the initiation of any antineoplastic therapy. The control group comprised 20 healthy children enrolled in the ‘PICTURE’ study. Inclusion criteria for controls required structural matching by age and sex, and a verified healthy status based on routine physical and biochemical examination. Exclusion criteria for the control group included any history of neoplastic or chronic metabolic diseases (e.g., diabetes), chronic autoimmune conditions, recent acute infections (within 4 weeks prior to sampling), and the recent use of medications known to significantly alter metabolic profiles, such as systemic corticosteroids or antibiotics. Clinical data and biospecimens were collected in accordance with approvals granted by the Bioethics Committee at the Wroclaw Medical University (ref: KB-667/2019 and KB525/2021). The data utilized, including both the patient and healthy control cohorts, were derived from research conducted under these institutional approvals, with biological samples collected between 2021 and 2023.

### 2.2. Sample Preparation

Peripheral venous blood was collected into calcium-balanced, lithium-heparinized tubes. The use of lithium heparin is standard practice in clinical metabolomics due to its minimal interference with most endogenous metabolite profiles compared to other common anticoagulants, which can significantly alter the metabolic signature. Blood samples were centrifuged at 2000× *g* for 15 min at room temperature, and the plasma was aliquoted and immediately cryopreserved at −76 °C; the interval between venipuncture and freezing was maintained below 30 min to minimize ex vivo metabolic drift. Cerebrospinal fluid (CSF) samples were acquired under strictly aseptic conditions and centrifuged at 1500× *g* for 15 min at 4 °C to remove cellular debris. Processed CSF was divided into 0.1 mL aliquots and stored at −76 °C. Prior to metabolomic profiling, samples were thawed on ice at 4 °C. While thawing at 20 °C was initially considered to ensure complete dissolution of solutes, we have transitioned to a 4 °C protocol to mitigate the risk of enzymatic degradation and thermal instability of labile metabolites. Single-use aliquots were employed to strictly avoid freeze–thaw cycles, thereby preserving the biochemical integrity of the biospecimens.

### 2.3. Sample Analysis

Metabolic profiling was performed using a Dionex Ultimate 3000RS UHPLC system (Thermo Fisher Scientific, Dreieich, Germany) coupled to a Bruker Compact ESI-QTOF mass spectrometer (Bruker Daltonics, Bremen, Germany) operating in positive and negative electrospray ionization modes. Chromatographic separation for cerebrospinal fluid and plasma samples analyzed on the BEH Amide column was achieved using an ACQUITY UPLC BEH Amide column (2.1 × 100 mm, 1.7 μm; Waters, Milford, MA, USA) equipped with a BEH Amide VanGuard pre-column (2.1 × 5 mm, 1.7 μm). The column temperature was maintained at 45 °C and the autosampler temperature was set at 4 °C with an injection volume of 5 μL.

For ESI+ analysis, water containing 0.1% formic acid and acetonitrile containing 0.1% formic acid were utilized as mobile phases. D4-Lys was added as an internal standard for signal normalization. For ESI− analysis, ammonium bicarbonate-based mobile phases (10 mM NH_4_HCO_3_, pH 9) were applied. The flow rate was 0.4 mL min^−1^ in both ionization modes. Mass spectra were acquired in Auto MS/MS mode over the *m*/*z* range of 20–1000 for ESI+ and 70–1000 for ESI−. Data-dependent fragmentation was performed using collision energies ranging from 20 to 60 eV in positive mode and from 25 to 35 eV in negative mode.

All sample preparation procedures were conducted at 4 °C using LC-MS grade reagents purchased from Merck. Biological samples were thawed on ice and homogenized for 5 min at 1200 rpm and 4 °C.

Regarding the extraction of cerebrospinal fluid, 100 μL of the material was mixed with 400 μL of a MeOH:ACN (1:1, *v*/*v*, −20 °C) mixture and 5 μL of D4-Lys. The mixture was vortexed for 3 min at 1200 rpm and 4 °C, incubated for 60 min at −20 °C, and centrifuged at 13,000 rpm for 15 min at 4 °C. A 200 μL aliquot of the supernatant was dried in a vacuum concentrator at 30 °C for 120 min. The residue was subsequently reconstituted in 100 μL of an H_2_O:MeOH:ACN (2:1:1, *v*/*v*) mixture, followed by ultrasonication and centrifugation at 13,000 rpm for 10 min at 4 °C.

For plasma samples, 100 μL of the material was combined with 300 μL of methanol (−20 °C) and 5 μL of D4-Lys. The samples were vortexed for 5 min at 1200 rpm at 4 °C, incubated for 60 min at −20 °C, and centrifuged at 15,000 rpm for 15 min at 4 °C. For BEH Amide column analysis, the supernatant was transferred directly to chromatographic vials. For HSS T3 column analysis, the remaining 200 μL of the supernatant was dried at 30 °C for 120 min and reconstituted in 100 μL of 0.1% (*v*/*v*) formic acid in 5% (*v*/*v*) methanol in water.

Quality control samples were prepared by pooling 10 μL aliquots from each biological sample to monitor instrument stability and analytical reproducibility. Raw LC-MS data were processed using MetaboScape 2021b (Bruker Daltonics, Germany).

### 2.4. Statistical Analysis

Subsequently, data pre-processing was performed using an in-house Python 3.14.4.-based workflow developed for untargeted metabolomics studies. The workflow included blank filtering, QC-based feature filtering, signal normalization, missing-value filtering, feature consolidation, logarithmic transformation, and generation of the final metabolite abundance matrix. Replicate injections were averaged at the patient level prior to statistical analysis. Statistical analysis.

Data pre-processing and comprehensive statistical analyses were performed using MetaboAnalyst 6.0 and Statistica 13.3 software (Wroclaw Medical University license). To eliminate systematic measurement errors, standardize variance, and ensure sample comparability, the raw metabolic profiling data were normalized centered and subjected to Pareto scaling prior to all multivariate analyses.

Univariate statistical analysis was performed to identify metabolites with differential abundances between plasma sample groups. Mann–Whitney U test was applied for each metabolite to compare normalized metabolites among groups. The *p*-values were computed and subsequently adjusted to the false discovery rate (FDR) method. In addition, Cliff’s delta was calculated as a non-parametric effect size to quantify the magnitude and direction of the difference between groups. Significant metabolites were identified by setting a threshold of FDR < 0.01 and |Cliff’s delta| ≥ 0.474.

Unsupervised PCA was initially conducted to evaluate the overall data structure, distribution, and natural clustering of the samples. Bootstrap-stability PCA was assessed using 1000 bootstrap iteration to assess variance-weighted contribution of metabolites across principal component, for CSF samples. To maximize the discrimination between the pre-B ALL patient group and the healthy control group, supervised OPLS-DA was applied. Furthermore, a PLS-DA biplot was utilized to determine the directionality of the metabolic changes. Additionally, significant metabolites were also identified by setting a VIP threshold above 1.5.

To evaluate the diagnostic potential and robustness of the identified metabolic profile, an SVM-RFE was implemented. Significant features distinguishing the study groups were identified using EBAM with a strict FDR threshold set at 0.011. Finally, to explore coordinated disruptions in biochemical pathways, hierarchical clustering analysis (visualized via dendrograms and heatmaps) and metabolite correlation matrices (Pearson’s correlation coefficient) were generated for both plasma and CSF samples.

MetaboAnalyst 6.0 was used for enrichment analysis, based on metabolites identified as significant from univariate and multivariate analyses. Metabolites selection criteria included thresholds from statistical analysis results, based on: FDR and Cliff’s delta, VIP scores and the metabolites from CSF samples that validated bootstrap-stability from PCA approach. Selected metabolites were mapped to biological pathways using the KEGG database. The threshold considered for a valid enrichment pathway was *p*-value of 0.05.

## 3. Results

### 3.1. Study Population

The study group comprised children newly diagnosed with acute pre-B lymphoblastic leukemia (ALL). All biological samples, including peripheral blood plasma and cerebrospinal fluid (CSF), were collected at the time of diagnosis, prior to the initiation of any systemic chemotherapy or supportive pharmacological interventions, as part of routine clinical diagnostic procedures. The control cohort for plasma analysis consisted of age- and sex-matched healthy volunteers. Structural matching was defined by identical distribution patterns for sex (χ^2^ test, *p* = 0.85) and age (median age 4.75 years; Mann–Whitney U test, *p* = 0.72) to minimize confounding effects, as metabolic profiles in pediatric populations are highly dynamic and age-dependent. Regarding the absence of CSF samples in the control group, this constitutes an inherent limitation of this pilot study, dictated by ethical considerations regarding invasive procedures in healthy pediatric subjects. Consequently, CSF metabolomic analysis was restricted to the ALL cohort to establish a baseline profile prior to treatment onset. Due to the high biological variability among children in this age range, dietary regimens were not standardized or recorded; furthermore, the acute nature of the clinical presentation and the immediate requirement for diagnostic sampling prior to treatment initiation precluded the collection of fasting-state metadata. Given the pilot nature of this study, these findings should be considered exploratory, with future research requiring larger, multicenter cohorts to validate these metabolic signatures.

### 3.2. Data Pre-Processing

In order to eliminate systematic measurement errors and ensure the comparability of samples, the raw metabolic profiling data were normalized. In addition, Pareto scaling was applied prior to conducting multivariate analyses. This step reduced the dominant influence of metabolites with the highest concentrations, whilst preserving the original data structure. Analysis of the distribution before and after pre-processing [Figure S1] indicated correct centering of the data and standardization of variance across samples, which provided the necessary foundation for conducting reliable multivariate statistical analyses.

### 3.3. Key Discriminatory Plasma Metabolites

Before exploring the overarching variance and structural data patterns through multivariate statistical models, we first present the specific metabolites that exhibited significant discriminatory power between the pediatric leukemia patients and the healthy controls. These key alterations, identified through rigorous univariate and multivariate statistical frameworks (detailed in subsequent sections), are systematically summarized in Table 1.

Overall, patients with pre-B ALL exhibited elevated levels of purine catabolites, specifically hypoxanthine and xanthine. Additionally, we observed increased concentrations of specific lipid species and other compounds, including oleamide, dulcitol, and phosphatidylcholine derivatives (e.g., PAF C16). Conversely, the leukemia cohort was characterized by a notable reduction in *L*-cysteine, *L*-2,4-diaminobutyric acid, prasterone sulfate, and 1,7-dimethylxanthine compared to the healthy control group. These changes collectively point toward an early, systemic dysregulation of purine, lipid, and amino acid/nitrogen metabolism, providing a biological foundation for the multivariate and pathway enrichment analyses that follow.

### 3.4. Plasma Data Analysis

**Figure 1:**
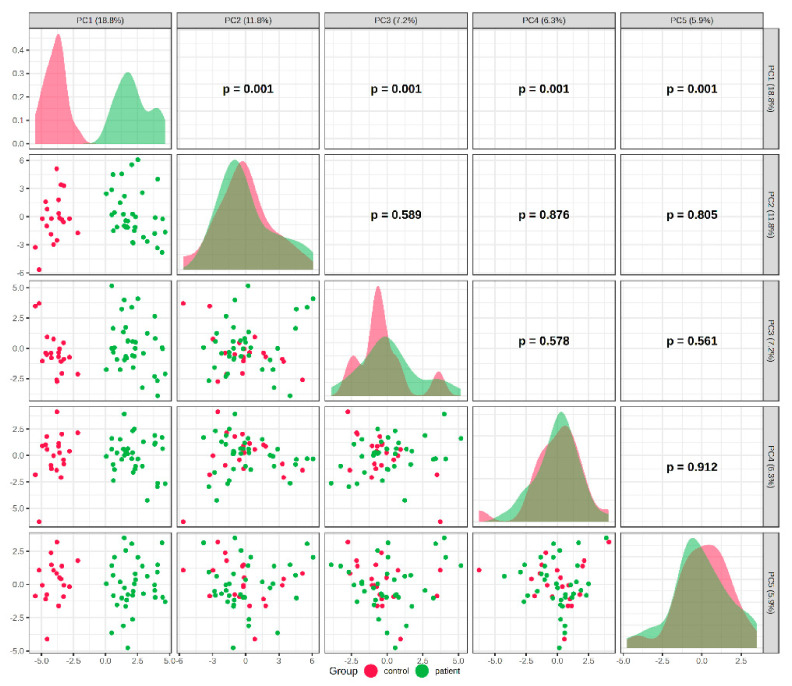
PCA plasma analysis.

To assess the overall structure of the data and the natural clustering of samples, a PCA was performed [Figure 1]. Scatter plots for the first five components (PC1–PC5) revealed that the largest proportion of variance in the dataset (PC1, 18.8%) showed substantial separation of the patient group from the control group (*p* = 0.001 for the PC1 distribution). Furthermore, an in-depth analysis in the PC1 and PC2 component space suggested the presence of heterogeneity within the study group. Two distinct subclusters of patients were identified, which may indicate the existence of distinct metabolic phenotypes or different stages of disease progression in the study cohort.

To maximize the separation between the patient group and the control group, the supervised OPLS-DA method was applied [Figure S2]. The Scores Plot for the OPLS-DA model supported complete discrimination between the two groups along the predictive component (T score [1], 17.6%), whilst also illustrating intra-group variability (Orthogonal T score).

To assess the diagnostic potential of the identified profile, an SVM-RFE was used [Figure S3]. This analysis showed that the highest classification accuracy (an error rate of just 3%) was achieved using the full panel of 76 variables. Reducing the number of metabolites used resulted in a systematic increase in error (up to 27.1% with 6 variables), which demonstrates that the observed phenotypic change is highly complex and multifactorial.

**Figure 2:**
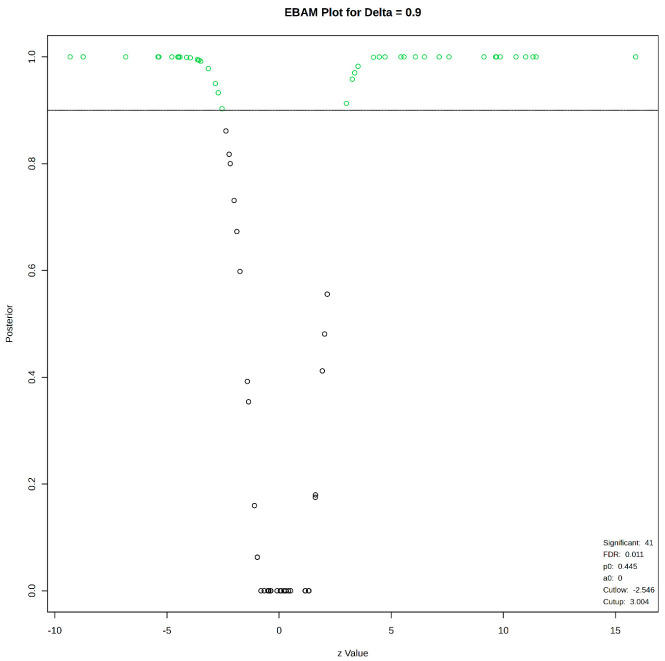
Plasma analysis based on the Empirical Bayesian Analytical Model (EBAM).

Empirical Bayesian analysis (EBAM) was used to identify the most statistically significant metabolites distinguishing the two groups [Figure 2]. This model identified 41 significant features (using a strict FDR threshold of 0.011).

**Figure 3:**
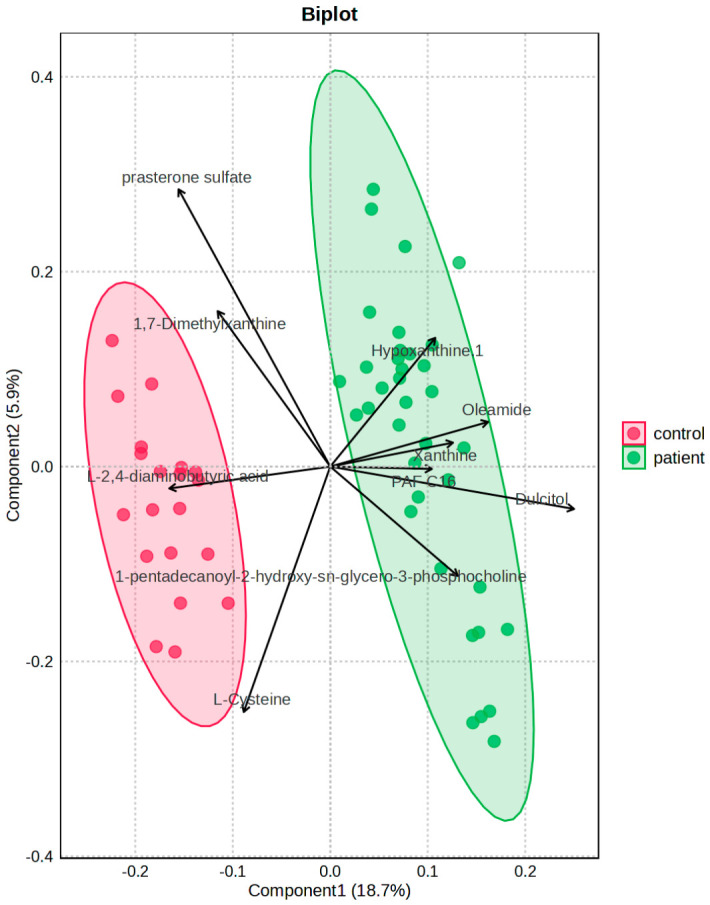
Biplot from the PLS-DA model plasma analysis.

The biplot from the PLS-DA model allowed the direction of these changes to be determined [Figure 3]. Changes in the metabolic profile in patients were mainly driven by elevated concentrations of compounds such as: Oleamide, Dulcitol, Hypoxanthine, Xanthine and selected phosphatidylcholine derivatives (e.g., PAF C16). In contrast, the control group was characterized by higher concentrations of prasterone sulphate, 1,7-dimethylxanthine and amino acids such as *L*-cysteine and *L*-2,4-diaminobutyric acid.

**Figure 4:**
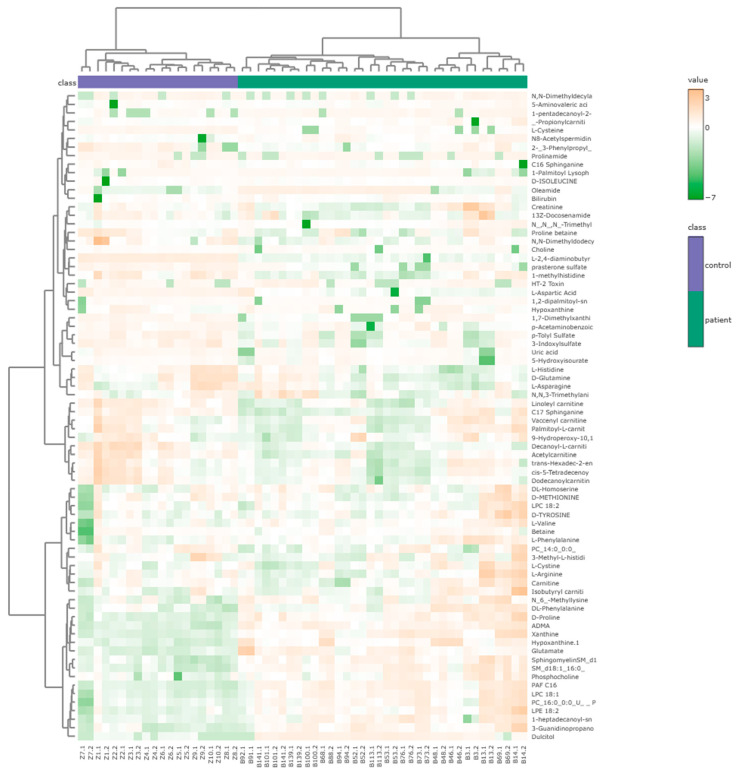
Heatmap with dendrogram for plasma analysis.

**Figure 5:**
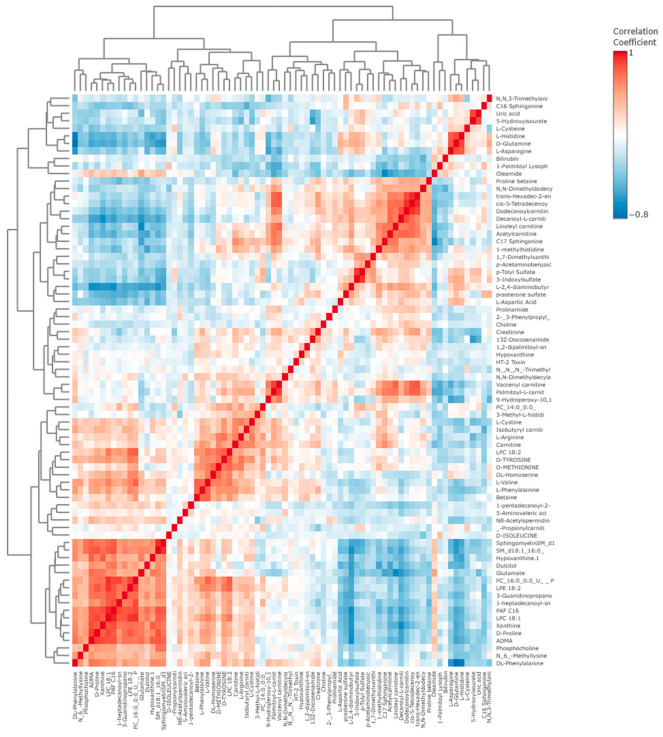
Plasma metabolites correlation matrix.

The final confirmation of the identification of a specific metabolic signature is provided by hierarchical clustering analysis [Figure 4]. The dendrogram superimposed on the heatmap of concentrations clearly divided the samples into two main clusters corresponding to the control group and the patients. A parallel metabolite correlation matrix revealed [Figure 5] the existence of strongly correlated (both positively and negatively) clusters of compounds, suggesting that the observed changes are not isolated fluctuations of individual metabolites, but reflect coordinated disturbances of entire biochemical pathways in patients.

### 3.5. CSF Metabolome

In this pilot study, we provide descriptive data on the CSF metabolome of ALL patients in the absence of a control group. While these findings are limited, they offer a baseline for future studies focusing on longitudinal changes during treatment regimens or therapy response, which will be the subject of our subsequent investigations.

PCA was therefore applied to the CSF metabolomic profile to investigate the main patterns of metabolic variability among ALL patients. PCA was used as a dimensionality reduction approach to summarize the dominant sources of variation across metabolites and to identify the metabolites contributing most strongly to patient-level metabolic heterogeneity. Based on the explained variance curve and the elbow-like reduction after the first components; the first four principal components were retained. Together, PC1–PC4 explained 64.18% of the total variance, indicating that these components captured the major metabolic structure of the dataset [Figure S4].

**Figure 6:**
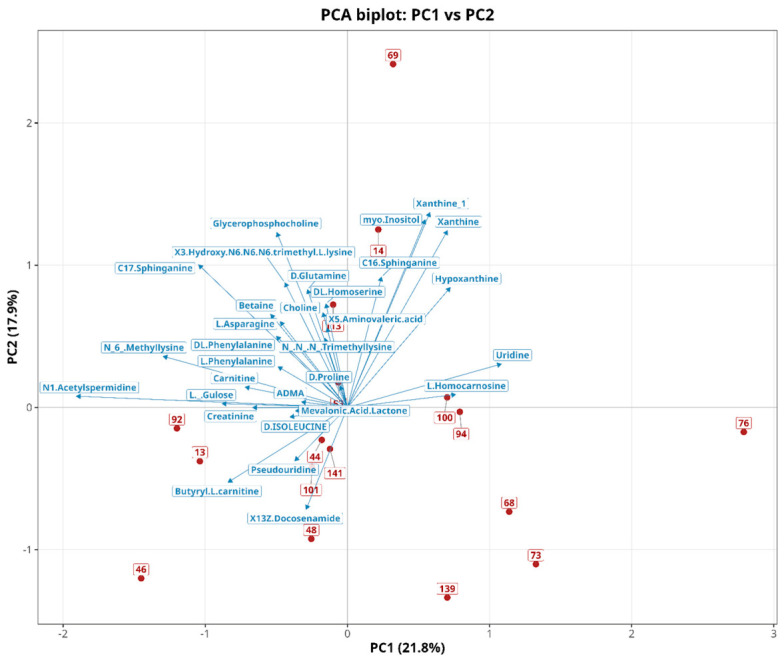
PCA biplot of PC1 versus PC2 in observed metabolites from CSF samples. Red dots represent individual patients and arrows represent metabolite loading vectors. Metabolites are labeled close to arrows. Arrow orientation indicates the contribution of metabolites with PC1 and PC2, whereas arrow length reflects their relative contribution strength to the variance captured within the PC1 and PC2 subspace.

Biplots were generated to jointly visualize patient distribution and metabolite loading directions from PC1 to PC4 [Figure 6]. Metabolites represented by longer arrows and located away from the origin were considered stronger contributors to the corresponding PCA plane, whereas patients positioned in the same direction as a metabolite vector were interpreted as being more strongly associated with that metabolic pattern.

Across the PCA biplots, several patients showed distinct positioning from the central cluster, suggesting intra-patient metabolic heterogeneity within the CSF cohort. In particular, biplots highlight throughout the four principal components the correlation observed metabolite-metabolite across each component. Metabolites were considered bootstrap-stable when their variance-weighted contribution across PC1–PC4 exceeded the expected contribution threshold in at least 75% of 1000 bootstrap iterations. C17-sphinganine, N6-methyllysine, butyrylcarnitine, uridine, myo-inositol, xanthine, and glycerophosphocholine were retained as stable contributors to the dominant metabolic variability observed.

Interestingly, C17-sphinganine was identified as one of the most stable PCA-derived contributors in the CSF patient-only dataset [Figure S5]. This observation may be biologically relevant because sphingolipid metabolism has previously been reported to be altered in acute leukemia, with increased plasma levels of sphinganine, sphingosine, and ceramide described in AML patients. This finding is consistent with previous observations reported in other malignancies [17]. Additionally, butyrylcarnitine was identified as a consistent metabolic contributor among CSF patients. In other cancer models, Li Y et al. reported that the metabolite has been implicated in proliferative processes in head and neck cancer (HNC) [18]. On breast cancer studies, elevated levels of circulating butyrylcarnitine are associated with increased cancer risk and progression [19].

Although its role in ALL remains poorly investigated, studies in other cancer models have suggested potential cancer-related relevance. Therefore, different reported studies faced limitations in establishing causal relationships due to case–control design limitations [18]. Subsequently, uridine was identified in pyrimidine metabolism associated with uracil and L-phenylalanine [20]. This pathway is associated with de novo pyrimidine synthesis, playing a role in cell growth and proliferation [21]. Lastly, glycerophosphocholine is related to phospholipid and choline metabolism and may reflect membrane turnover or cellular remodeling, possibly involved inflammatory processes [22,23]. Is it important to note that, due to study limitation related to case–control on CSF samples, new experimental and complete studies can lead to a correct and accurate and precise interpretation. All CSF metabolites were identified through unsupervised PCA and bootstrap-stability analysis and should therefore be interpreted as exploratory metabolic contributors rather than statistically confirmed biomarkers. Further validation in larger cohorts may help clarify the effects of these observations, especially in well-designed case–control experimentation. Following the descriptive analysis of significant metabolites in plasma and CSF samples, metabolites with available KEGG Compound IDs were selected for enrichment analysis. This resulted in 21 metabolites from the univariate plasma analysis, 12 metabolites from the multivariate plasma analysis, and 7 metabolites from the CSF metabolite set.

### 3.6. Enrichment and Pathways

#### 3.6.1. Identification of Significant Metabolic Pathways

**Figure 7:**
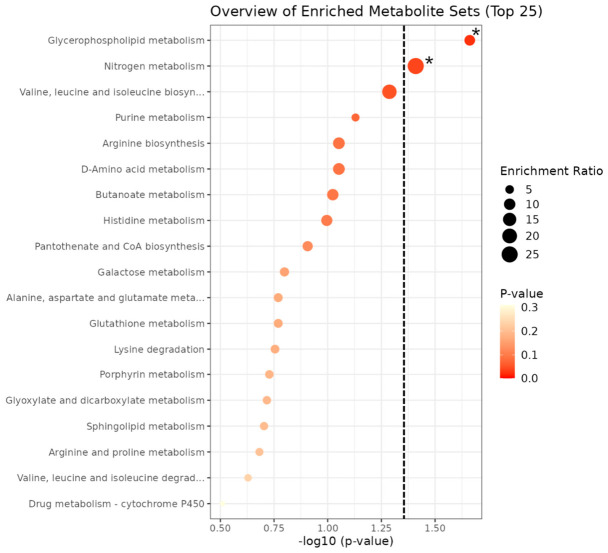
KEGG pathway enrichment analysis of plasma metabolites identified through univariate statistical analysis. Enrichment was performed on 21 significant metabolites. Circle size represents the enrichment ratio, color intensity indicates the *p*-value of metabolites involved in the metabolism pathways. The dashed line represents the *p*-value threshold (*p* = 0.05). The metabolism pathways marked with (*) correspond to *p*-value significance enrichments **(*p* < 0.05)**.

**Figure 8:**
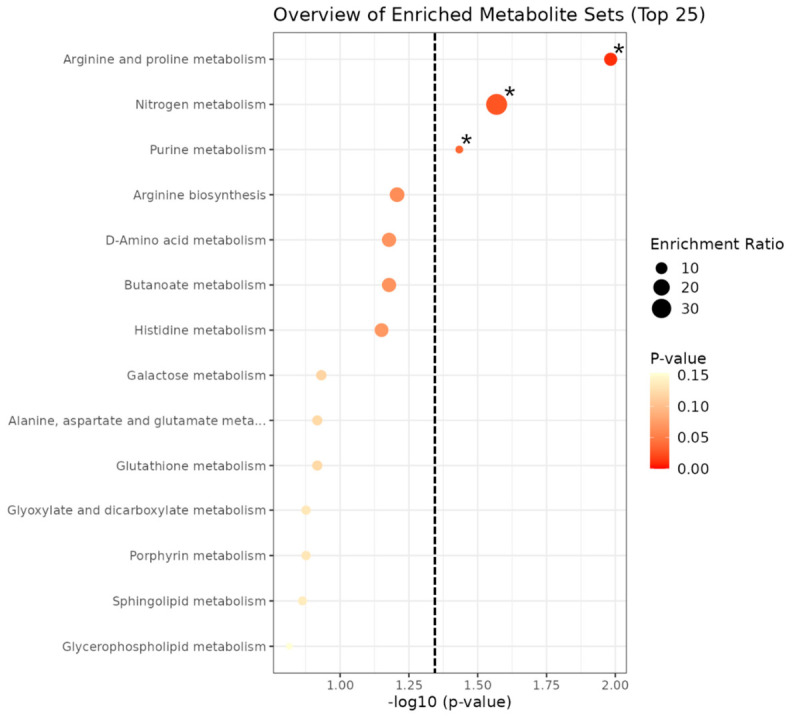
KEGG pathway enrichment analysis of plasma metabolites identified through multivariate statistical analysis. Enrichment was performed on 12 significant metabolites from the multivariate analysis. Circle size represents the enrichment ratio, color intensity indicates the *p*-value of metabolites involved in the metabolism pathways. The dashed line represents the *p*-value threshold (*p* = 0.05). The metabolism pathways marked with (*) correspond to *p*-value significance enrichments **(*p* < 0.05)**.

Pathways identified from the enrichment analysis did show some overlap across the different statistical approach methodologies. Due to differences in thresholds measurements, two significant pathways were identified from the univariate statistical approaches: Glycerophospholipid metabolism and Nitrogen Metabolism. On the other hand, three significant pathways were also identified from the multivariate statistical approach: Arginine and Proline metabolism, Nitrogen metabolism, and Purine metabolism [Figure 7 and Figure 8]. An overlap of a metabolic pathway has been observed “Nitrogen Metabolism”, indicating reproducible signals independently from the statistical approach. Therefore, no significant metabolic pathways were identified from the CSF metabolites set, suggesting possibilities of performing differential statistical analysis, including differentiated treatment to extract significant metabolites.

#### 3.6.2. Glycerophospholipid, Arginine and Proline, Purine and Nitrogen Metabolism

Acute Lymphoblastic Leukemia (ALL) cells alter metabolic process of arginine and proline to survive and grow rapidly, a process categorized as metabolic rewiring [24]. In B-ALL, the adipocyte-rich microenvironment has been reported to promote chemoresistance by supplying or inducing metabolic programs that support leukemic cell fitness under therapeutic stress [15]. Notably, in the presence of methotrexate, adipocyte-derived signals activate arginine biosynthesis together with alanine, aspartate, glutamate, and glutathione metabolism, providing protection against oxidative stress and chemotherapy-mediated cytotoxicity. Therefore, the enrichment of arginine and proline metabolism in our results may reflect amino-acid metabolic adaptation rather than a single isolated metabolite change. This is relevant because arginine and proline metabolism connects several related metabolites, including glutamate and ornithine, which may contribute to nitrogen balance and biosynthetic demand. Then, ornithine, with its capability of inhibiting isoforms, proceeds to rewire proline metabolism, which automatically impacts the arginine biosynthesis [15,25].

Additionally, similarly to the previously investigated metabolite pathways, the purine metabolism was identified from the multivariate statistical method. However, purine metabolism has been reported from AML studies as one of the significant criteria of survivability, as higher levels of related purine metabolism enzyme found in AML cohorts are associated with poorer survival outcome [26]. This recent published study suggests that AML therapies should be oriented toward targeting this metabolic pathway to improve therapeutic outcomes. Lastly, Nitrogen metabolism can possibly be relevant in ALL because proliferating leukemic lymphoblasts require a continuous supply of nitrogen-containing metabolites for amino-acid, protein, and nucleotide biosynthesis [27]. Hence, the signal of the enrichment was consistent on both statistical methods [Figure 7 and Figure 8]. In this context, glutamine is particularly important because it serves as a major nitrogen donor for purine and pyrimidine synthesis, linking nitrogen metabolism to cellular proliferation [28]. This interpretation aligns with the recognized therapeutic importance of amino-acid metabolism in ALL, exemplified by *L*-asparaginase treatment, which depletes extracellular asparagine and thereby exploits the reliance of leukemic cells on exogenous amino-acid availability [29].

### 3.7. Integration of Plasma and CSF Metabolomic Profiles

An investigation into the metabolic coupling between the systemic circulation and the central nervous system (CNS) involved the generation of a comprehensive correlation matrix. This matrix evaluated the top-performing plasma biomarkers against stable metabolites quantified within the cerebrospinal fluid (CSF). Subsequent statistical analyses elucidated a significant positive correlation between systemic plasma hypoxanthine and CSF glycerophosphocholine (r = 0.40, *p* < 0.05). Moreover, parallel evaluations indicated a robust positive association between systemic and CNS concentrations of xanthine (r = 0.51, *p* < 0.05).

These findings suggest that pathological metabolic perturbations, most notably those governing purine catabolism and phospholipid turnover, transcend the systemic circulation, manifesting functional counterparts within the privileged microenvironment of the CNS. The elevated accretion of these specific metabolites ostensibly mirrors the hyperactive nucleic acid turnover and aggressive proliferative dynamics intrinsic to leukemic blasts, a process mandating profound global metabolic reprogramming.

The metabolic concordance observed between plasma and CSF signatures implies that systemic biochemical shifts may influence the CNS metabolic environment. This dynamic highlights the pervasive systemic burden of leukemic progression on both blood–brain barrier (BBB) integrity and the broader neurochemical milieu. Furthermore, this metabolic coupling suggests a potential association between peripheral blood and the CNS metabolic environment. However, as these are preliminary observations from an exploratory pilot study, further validation in larger prospective cohorts is required to determine whether plasma metabolomics could serve as a surrogate marker for monitoring CNS involvement.

## 4. Discussion

The comprehensive metabolomic analysis of plasma and cerebrospinal fluid (CSF) in children with newly diagnosed pre-B acute lymphoblastic leukemia (ALL) provides preliminary evidence of profound, systemic metabolic reprogramming occurring in the patients’ organisms prior to the initiation of any therapeutic intervention. By securing all biological samples during standard diagnostic procedures before the start of chemotherapy induction, we minimized the confounding xenobiotic impact of cytostatic drugs and glucocorticoids, thereby enabling an objective characterization of the primary metabolic phenotype of ALL and providing direct insight into the biochemical essence of leukemogenesis [4,30]. The application of advanced mass spectrometry platforms featuring high analytical resolution, combined with a rigorous statistical apparatus including both unsupervised methods and supervised machine learning algorithms, enabled the identification of candidate metabolic signatures associated with the pre-B ALL state, which clearly and multidimensionally differed from the healthy control group. The obtained results indicate that the development of ALL appears to be associated with extensive metabolic reprogramming of fundamental metabolic pathways responsible for energy homeostasis, purine metabolism, lipid biosynthesis and degradation, as well as amino acid turnover, reflecting the specific bioenergetic and biosynthetic demands of rapidly proliferating neoplastic blasts [31,32].

The principal component analysis performed on the plasma suggested a clear spatial separation between the patient cohort and the demographically matched control group, with the first principal component explaining a significant percentage of the total variance. Simultaneously, a detailed exploration of the component space revealed distinct heterogeneity within the leukemia group, manifesting as a division of the patient population into two autonomous subclusters. This observation suggests the existence of at least two distinct metabolic phenotypes among children with pre-B ALL, which may implicate different pathways of disease progression, varying dynamics of subclinical tumor advancement, or differential sensitivity to microenvironmental signals, despite the absence of discernable differences in routine clinical stratification. While similar metabolic resistance and heterogeneity have been reported in bone marrow niches of adult patients, this work provides one of the first pieces of evidence of such cellular diversity reflected directly in the metabolic profile of pediatric peripheral blood [4,31].

Among the strongest markers determining group separation in statistical models such as OPLS-DA or EBAM, a significant increase in plasma concentrations of hypoxanthine and xanthine stood out in children with ALL [2]. These compounds represent key end-metabolites of the purine degradation pathway, and their accumulation serves as a direct marker of accelerated nucleic acid turnover and intense, uncontrolled proliferation of leukemic blasts leading to increased cell breakdown and the activation of purine catabolism [31,32]. Such activation may represent an evolutionary metabolic adaptation of cancer cells, optimizing energy management and nucleotide resources under conditions of permanent replicative stress [30,32]. In opposition to these changes, a notable decrease in the levels of *L*-cysteine and *L*-2,4-diaminobutyric acid was recorded in the plasma of patients with ALL. Cysteine is a sulfur-containing amino acid acting as a fundamental precursor in the biosynthesis of glutathione, the primary intracellular antioxidant defense system. The depletion of its reserves points toward powerful oxidative stress accompanying the development of leukemia and highlights the critical dependence of blasts on cellular defense mechanisms against reactive oxygen species, revealing potential new targets for metabolically directed therapies [33,34]. Furthermore, the amino acid metabolism abnormalities observed are consistent with the concept of metabolic reprogramming of cancer cells, involving increased utilization of amino acids as biosynthetic substrates [29,35]. The implementation of the advanced SVM-RFE feature selection algorithm suggested that the highest diagnostic classification accuracy was achieved exclusively when taking the full panel of metabolites into account, confirming the multifactorial and highly complex nature of metabolic disturbances in ALL. This result suggests that single, isolated biomarkers are insufficient for precise diagnostic modeling, whereas multi-component predictive algorithms exhibit incomparably greater clinical value.

In the healthy control group, prasterone sulfate (DHEA-S), 1,7-dimethylxanthine (paraxanthine), and the amino acids *L*-cysteine and *L*-2,4-diaminobutyric acid were predominant. Low DHEA-S concentrations in patients may indicate a primary inhibition of the hypothalamic–pituitary–adrenal axis even before the start of therapy, an occurrence previously associated primarily with the exogenous effects of glucocorticoid use [36,37]. This observation suggests the possibility that the malignancy itself might impair adrenal steroid synthesis. A key component of this study was a parallel, comprehensive multi-platform analytical workflow for the detailed characterization of the CSF metabolome. To date, most metabolomic studies in ALL have focused on peripheral blood or bone marrow [4,6], while the CSF matrix has been largely overlooked, despite the fact that the central nervous system (CNS) represents one of the most frequent and anatomically challenging niches for leukemic relapse. Correlation analyses performed in the CSF of children with pre-B ALL revealed strong, coordinated linear dependencies between hypoxanthine and xanthine (r = 0.512) as well as between choline and glycerophosphocholine (r = 0.402). This provides preliminary evidence that the pathological disturbances in purine and phospholipid metabolism are not restricted to systemic circulation but exhibit faithful, functional counterparts within the unique microenvironment of the CNS. Furthermore, the strong positive correlation between carnitine and butyryl-*L*-carnitine (r = 0.486) indicates profound disturbances in mitochondrial transport and beta-oxidation of long-chain fatty acids, likely underlying the systemic catabolic state observed in ALL [38]. An intriguing phenomenon was the strong negative correlation between 13Z-docosamide and the vast majority of amino acids. Docosamides are derivatives of erucic acid recently linked to the regulation of the inflammatory response in the CNS [39,40]; hence, their elevated concentration in the CSF may reflect an ongoing, subclinical inflammatory process triggered by leukemic cells even prior to the clinical manifestation of symptomatic leukostasis.

The topological analysis based on the dendrogram of CSF profiles allowed for the precise isolation of independent cluster architectures. The first major node grouped amino acids and beta-oxidation compounds, the second included nucleoside derivatives, while sphinganines formed a completely separated, autonomous branch. Such a topological pattern suggests that simultaneous but independent deregulation of protein, nucleic acid, and lipid metabolism occurs in the CSF of children with ALL, with the sphingolipid pathway potentially being disrupted independently of the others [22]. Disturbances in the sphingolipid pathway deserve special attention because these molecules determine the physicochemical structure of lipid rafts in cell membranes and mediate signaling cascades that induce apoptosis, and their abnormal metabolism has been repeatedly linked to the development of cancer cell resistance to chemotherapeutic agents within the CNS [41,42].

From a cell biology perspective, the identified metabolic signature directly reflects the fundamental pathogenic requirements of rapidly proliferating leukemic blasts [35,43]. The significant accumulation of purine catabolites, specifically hypoxanthine and xanthine, underscores the massive nucleic acid turnover and the hyperactivation of purine salvage pathways necessary to sustain constant DNA and RNA synthesis during uncontrolled cell division [44,45]. Concurrently, the upregulation of phosphatidylcholine derivatives points toward accelerated membrane biogenesis and the active remodeling of the lipid bilayer [46]. This lipid rewiring is critical not only for physical blast expansion but also for the maintenance of oncogenic signaling cascades, which are frequently anchored in membrane lipid rafts. Furthermore, the marked depletion of *L*-cysteine highlights a profound cellular vulnerability related to redox homeostasis; as leukemic cells generate high levels of reactive oxygen species (ROS) due to their hypermetabolic state, they continuously deplete systemic cysteine pools to synthesize glutathione and evade apoptosis [47]. Collectively, these systemic shifts are not merely downstream byproducts, but direct functional readouts of the altered cell biology driving pre-B ALL leukemogenesis [35].

It is essential to acknowledge that this work represents an exploratory pilot study. The modest sample size (19 ALL patients and 20 controls) necessitates a cautious interpretation of the findings. While the consistency of the observed metabolic signals across multiple analytical platforms and statistical models strengthens the reliability of the data, the small cohort size inherently limits the statistical power of the analysis and the generalizability of the results to the broader population of children with ALL. Furthermore, the cross-sectional nature of this study precludes the assessment of longitudinal metabolic shifts. Future research should prioritize the recruitment of larger, multi-center patient cohorts to validate these findings and facilitate the integration of metabolomic data with genomic and proteomic analyses within a multi-omic framework. Such efforts are crucial to determine whether the identified metabolic profiles can reliably predict treatment response, the risk of relapse, or the occurrence of specific therapy-related adverse effects, ultimately advancing the potential of mass spectrometry-based metabolomics as a tool for precision diagnostics and personalized medicine in pediatric oncology.

## Figures and Tables

**Table 1:** Metabolites differentiating leukemia patients from healthy controls.

Metabolite	Trend (ALL vs. Control)	Statistical Method(s)	Associated Pathway(s)
Hypoxanthine	Increased	EBAM, PLS-DA	Purine metabolism
Xanthine	Increased	EBAM, PLS-DA	Purine metabolism
Oleamide	Increased	PLS-DA	-
Dulcitol	Increased	PLS-DA	-
Phosphatidylcholine derivatives (e.g., PAF C16)	Increased	PLS-DA	Glycerophospholipid metabolism
*L*-Cysteine	Decreased	PLS-DA	Nitrogen metabolism, Glutathione metabolism
*L*-2,4-diaminobutyric acid	Decreased	PLS-DA	-
Prasterone sulfate	Decreased	PLS-DA	-
1,7-Dimethylxanthine	Decreased	PLS-DA	Purine metabolism

## Data Availability

The original contributions presented in this study are included in the article/Supplementary Materials. Further inquiries can be directed to the corresponding authors.

## Supplementary Materials

The following supporting information can be downloaded at: https://www.mdpi.com/article/10.3390/cells15141255/s1, Figure S1: Plasma metabolites standardization results.; Figure S2. OPLS-DA plasma analysis; Figure S3. Support Vector Machine (SVM) algorithm with Recursive Feature Elimination (RFE) for plasma analysis; Figure S4: PCA explained variance; Figure S5: Bootstrap stability of PCA CSF metabolites; Table S1: PMR Correlation.
