# Supplementary material for: Exploratory Metabolomic Profiling of Plasma and Cerebrospinal Fluid in a Pilot Study of Children with Acute Lymphoblastic Leukemia
Source: Cells. 2026 Jul 12;15(14):1255. doi: 10.3390/cells15141255 (PMC13406799; doi:10.3390/cells15141255)
Supplement: Supplementary file 1 [file cells-15-01255-s001.zip › Supllementary/Supplementary Figures.pdf]

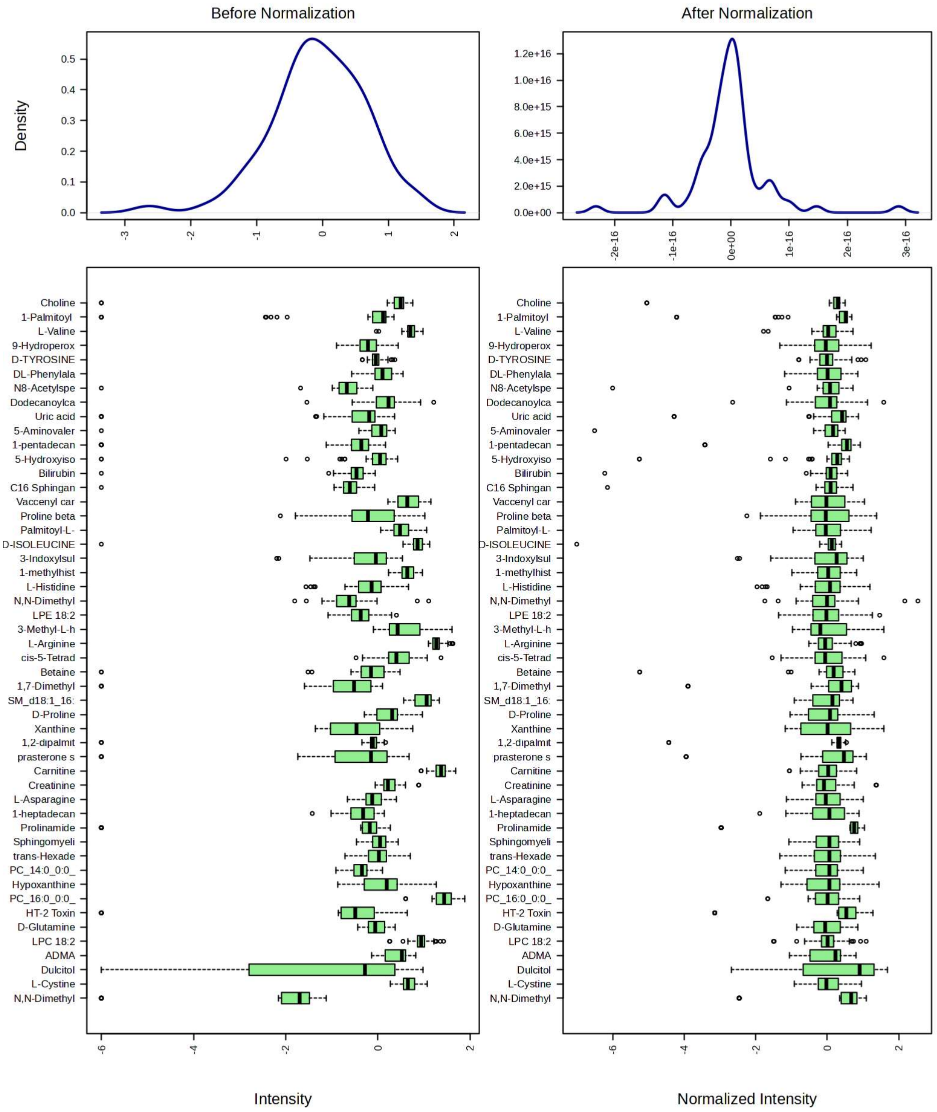

Figure S1. Plasma metabolites standardization results.

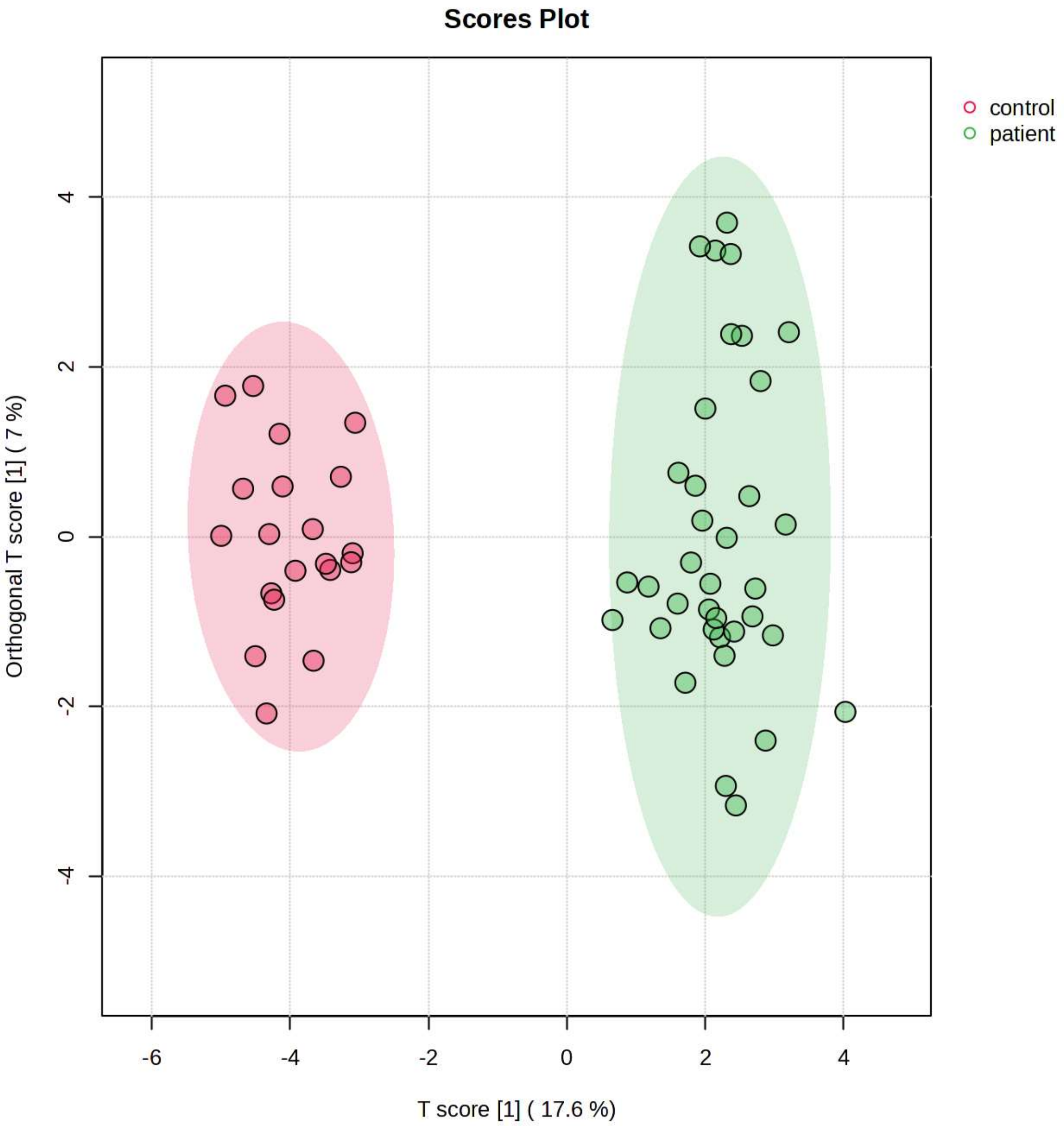

Figure S2. OPLS-DA plasma analysis.

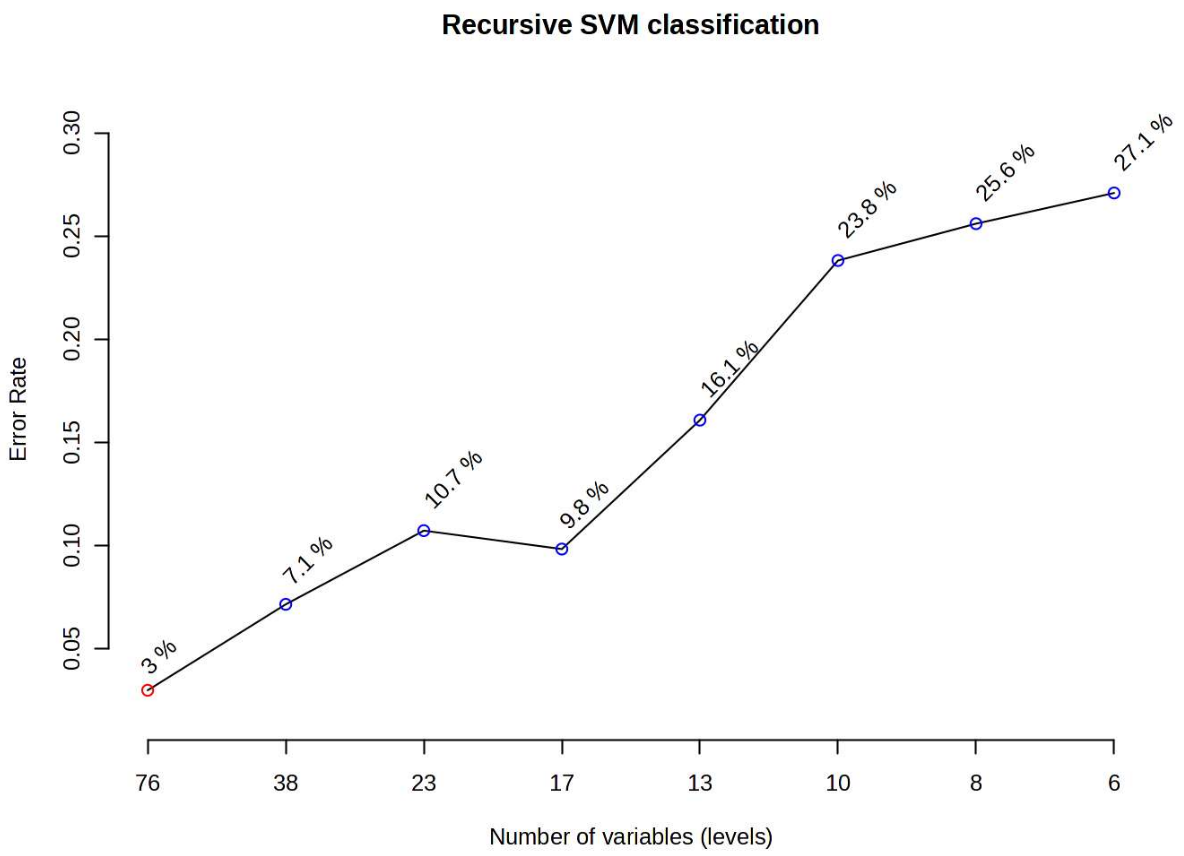

Figure S3. Support Vector Machine (SVM) algorithm with Recursive Feature Elimination (RFE) for plasma analysis.

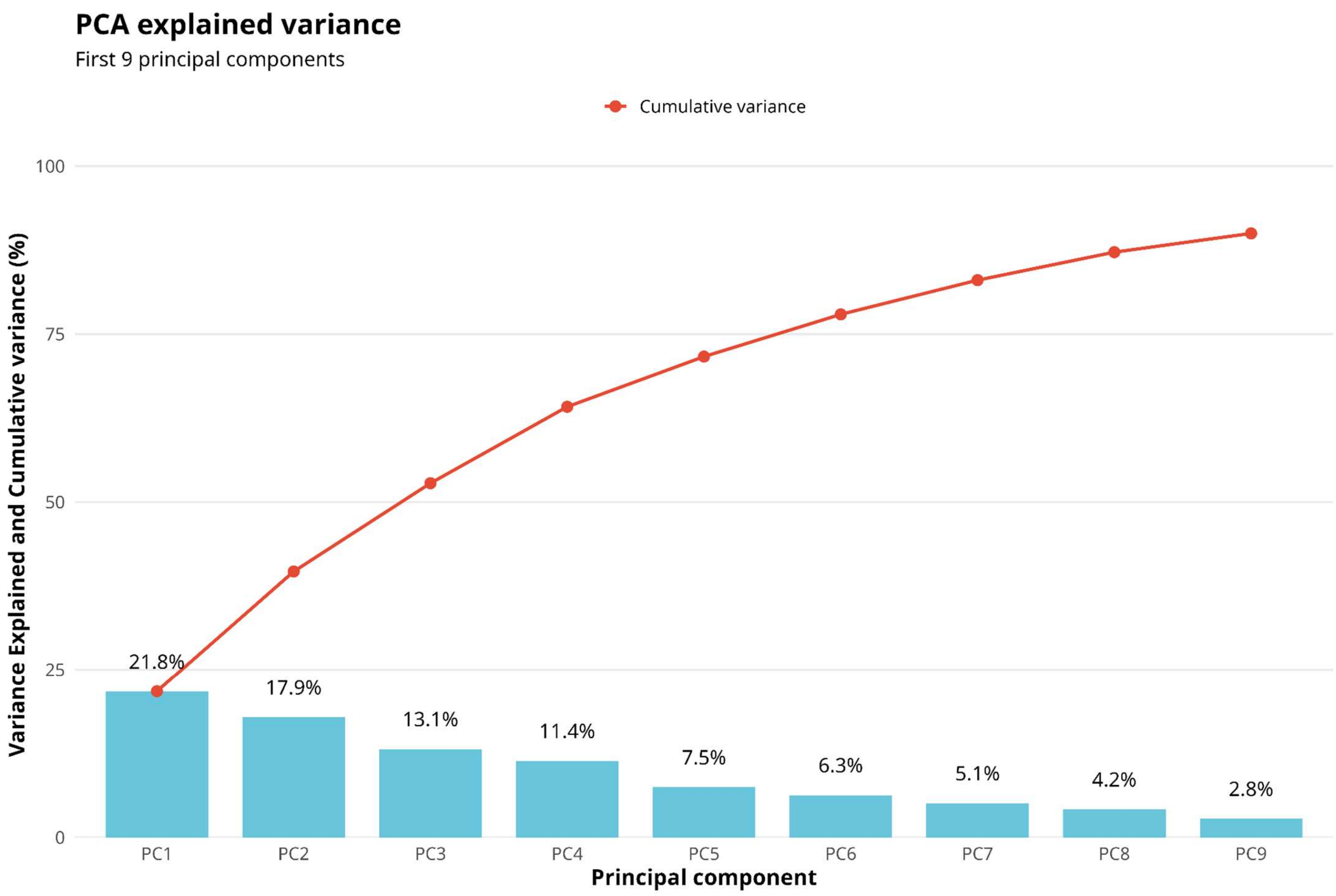

Figure S4. Explained and cumulative variance across the first nine principal components (PCs) in CSF samples. Blue bars represent the percentage of variance explained by each principal component, while the values above the bars indicate the corresponding explained variance percentages. The red line represents the cumulative explained variance across principal components. PCs 1–4 were retained, accounting for approximately 64% of the total variance.

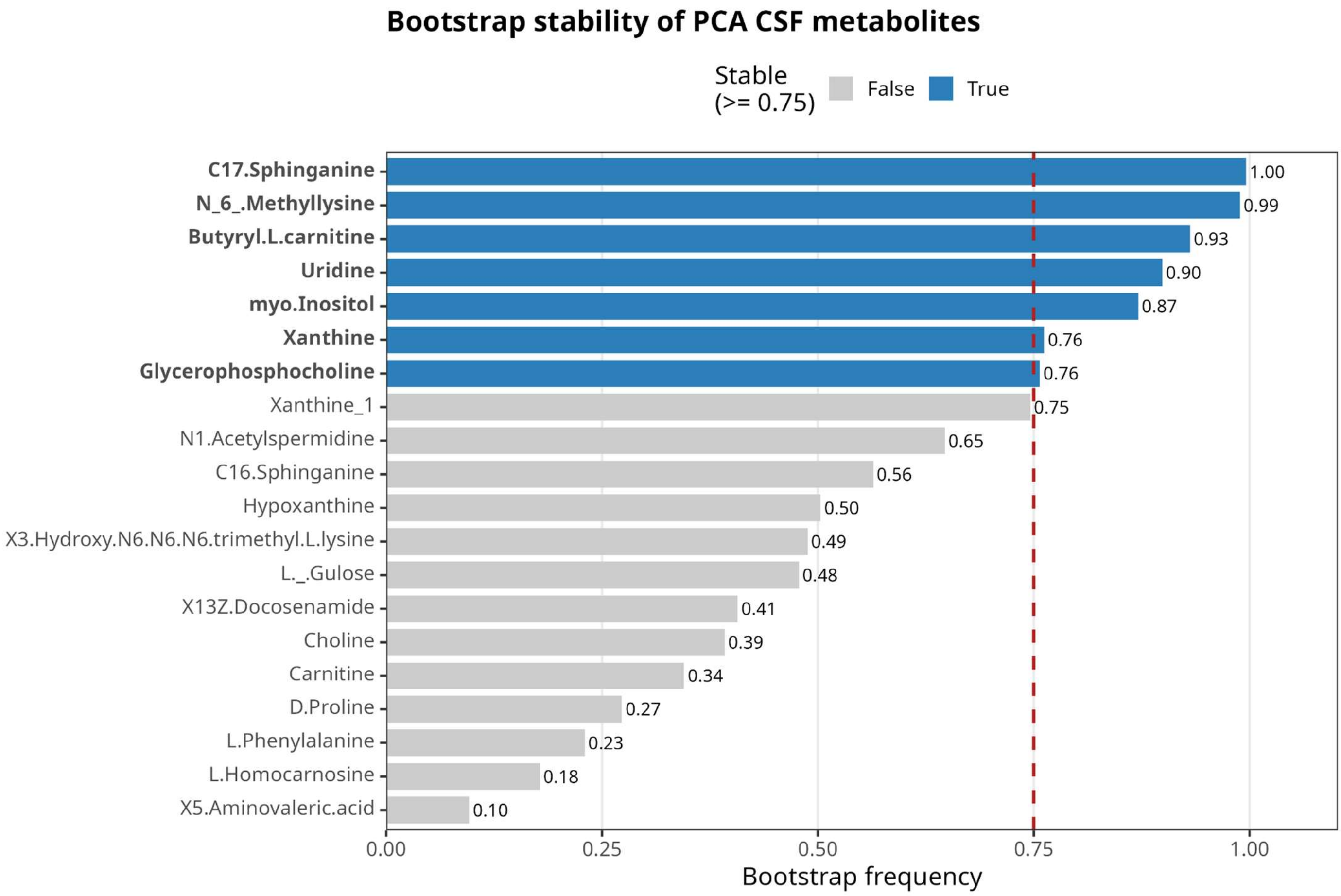

Figure S5. Bootstrap stability of PCA-derived metabolite drivers in the CSF in all patients. Bars represent the proportion of bootstrap iterations in which each metabolite exhibited a variance-weighted contribution above the expected threshold across PC1 to PC4. The red dashed line indicates the predefined stability threshold of 0.75 (metabolites selected in at least 75% of bootstrap iterations). Metabolites exceeding this threshold were considered bootstrap-stable PCA contributors, coloured in blue bars if bootstrap-stable, while metabolites below the threshold are shown in grey.
